# DAMA: a method for computing multiple alignments of protein structures using local structure descriptors

**DOI:** 10.1093/bioinformatics/btab571

**Published:** 2021-08-16

**Authors:** Paweł Daniluk, Tymoteusz Oleniecki, Bogdan Lesyng

**Affiliations:** Bioinformatics Laboratory, Mossakowski Medical Research Centre, Polish Academy of Sciences, 02-106 Warsaw, Poland; College of Inter-Faculty Individual Studies in Mathematics and Natural Sciences, University of Warsaw, 02-089 Warsaw, Poland; Department of Biophysics, Faculty of Physics, University of Warsaw, 02-093 Warsaw, Poland

## Abstract

**Motivation:**

The well-known fact that protein structures are more conserved than their sequences forms the basis of several areas of computational structural biology. Methods based on the structure analysis provide more complete information on residue conservation in evolutionary processes. This is crucial for the determination of evolutionary relationships between proteins and for the identification of recurrent structural patterns present in biomolecules involved in similar functions. However, algorithmic structural alignment is much more difficult than multiple sequence alignment. This study is devoted to the development and applications of DAMA—a novel effective environment capable to compute and analyze multiple structure alignments.

**Results:**

DAMA is based on local structural similarities, using local 3D structure descriptors and thus accounts for nearest-neighbor molecular environments of aligned residues. It is constrained neither by protein topology nor by its global structure. DAMA is an extension of our previous study (DEDAL) which demonstrated the applicability of local descriptors to pairwise alignment problems. Since the multiple alignment problem is NP-complete, an effective heuristic approach has been developed without imposing any artificial constraints. The alignment algorithm searches for the largest, consistent ensemble of similar descriptors. The new method is capable to capture most of the biologically significant similarities present in canonical test sets and is discriminatory enough to prevent the emergence of larger, but meaningless, solutions. Tests performed on the test sets, including protein kinases, demonstrate DAMA’s capability of identifying equivalent residues, which should be very useful in discovering the biological nature of proteins similarity. Performance profiles show the advantage of DAMA over other methods, in particular when using a strict similarity measure *Q*_C_, which is the ratio of correctly aligned columns, and when applying the methods to more difficult cases.

**Availability and implementation:**

DAMA is available online at http://dworkowa.imdik.pan.pl/EP/DAMA. Linux binaries of the software are available upon request.

**Supplementary information:**

Supplementary data are available at *Bioinformatics* online.

## 1 Introduction

Structures of proteins are conserved more than their sequences. It is believed, that methods based on analysis of structure, rather than sequence analysis, provide more complete information on residue conservation in evolutionary processes. This knowledge is crucial for both, determination of evolutionary relationships between proteins, and for the identification of structural patterns involved in similar functions. Unfortunately aligning structures is a computationally much more demanding task than aligning sequences. Nevertheless, structure alignment is commonly used in protein classification (e.g. Fox *et al.*, 2014; Orengo *et al.*, 1997) or in structural motif recognition (Singh and Saha, 2003). One of the most challenging tasks in computational biology is multiple structure alignment (MStA). There are several methods for computing MStA, like CBA (Ebert and Brutlag, 2006), Matt (Menke *et al.*, 2008), MASS (Dror *et al.*, 2003), MAMMOTH-Multi (Lupyan *et al.*, 2005), MultiProt (Shatsky *et al.*, 2004), MUSTANG (Konagurthu *et al.*, 2006) or POSA (Ye and Godzik, 2005). Some of them were reviewed in (Berbalk *et al.*, 2009). Since then some new methods have been developed, like MAPSCI (Ilinkin *et al.*, 2010), MISTRAL (Micheletti and Orland, 2009), 3DCOMB (Wang *et al.*, 2011), msTALI (Shealy and Valafar, 2012), mTM-align (Dong *et al.*, 2018) or Caretta (Akdel *et al.*, 2020).

The MStA problem can be formulated in several ways and existing algorithms differ by the type of alignments they compute. We distinguish methods in which structures are treated as rigid bodies (CBA, 3DCOMB) from methods that allow a certain level of flexibility (Matt, POSA, MASS). Some algorithms may allow for circular permutations (and other rearrangements) (MASS).

Most approaches either gradually join pairwise structural alignments into one MStA (i.e. perform progressive alignment), or align all structures simultaneously.

DAMA has been designed to bridge a gap between these approaches and ensure robust performance while imposing the least constraints on the solution. Quality of alignments is achieved by ensuring that the entire molecular environment of the aligned residues is taken into account. There are no constraints on the global superposition, which enables alignment of structures with significant spatial distortions (e.g. a different arrangement of domains connected by a flexible linker). Circular permutations and segment swaps are allowed as well. The concept of progressive alignment is applied in the initial phase for generating several alignments to be further improved by an evolutionary algorithm. Selected algorithms have already been implemented and optimized for CUDA graphical processors (Daniluk *et al.*, 2019).

## 2 Approach

**Fig. 1.**
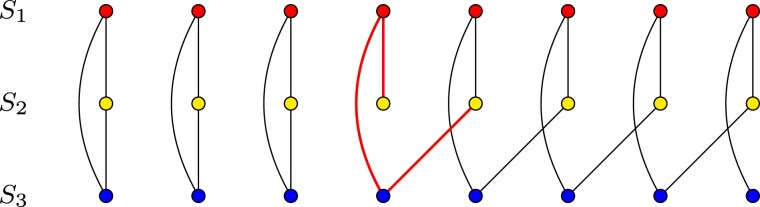
Example of inconsistency between pairwise alignments. Each row of dots presents residues in a protein structure. Arcs between dots denote pairwise alignment between residues. Three alignments between structures S1, S2 and S3 cannot be combined into a single multiple alignment. A gap at position 4 in S2—S3 causes ambiguity which makes several residues in e.g. S2 transitively (*via* S1 and S3) aligned

Finding optimal multiple alignments is a computationally difficult problem. However, it has been proven that a seemingly simpler problem of computing a multi-alignment as a consensus of given pairwise alignments is intractable as well (Daniluk and Lesyng, 2014). This is due to the fact, that not all sets of pairwise alignments may constitute a valid multiple alignment. It is disallowed for two different residues from a single structure to belong to the same column in the alignment. Nevertheless, it is easy to construct a set of pairwise alignments which would lead to such a condition if merged into a multiple alignment (see Fig. 1). Therefore, computing a multiple alignment from a set of pairwise alignments would require identifying all such conflicts, and finding an optimal way of removing them.

In this study, we tackle the multi-alignment problem in its most generic form. We aim at finding multiple alignments that may contain circular permutations, segment swaps and other sequential rearrangements, as well as, structural deformations.

**Fig. 2.**
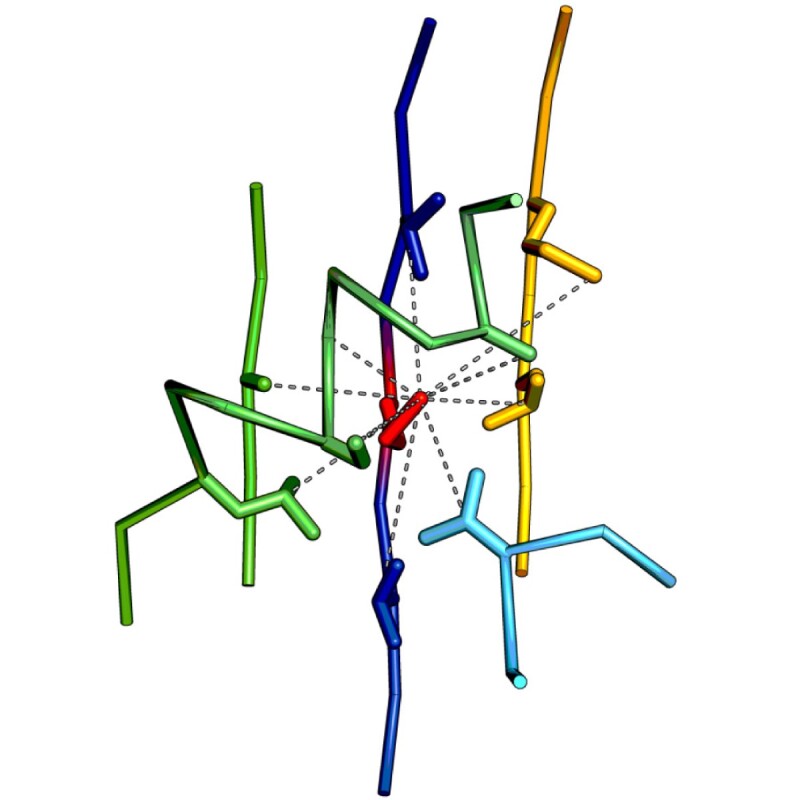
An exemplary descriptor built around the residue MET70 of 1lg7A contains nine contacts (dashed lines) between its central amino acid (red) and residues forming the centers of its elements. Some of the elements overlap forming longer segments [in particular fragments of two β-strands (blue and yellow) and a fragment of an *α*-helix (green)]. Altogether, this descriptor consists of five continuous segments

The only constraint we enforce on the multiple alignments is the similarity of local physico-chemical environments, which are characterized using molecular fragments called local descriptors of protein structure (Daniluk and Lesyng, 2011b; Hvidsten *et al.*, 2009). A local descriptor is a small part of a structure that can be viewed as a residue-attached local environment. In principle, it is possible to build a descriptor for every residue of a given protein. This process begins by identifying all residues in contact with the descriptor’s central residue. Elements are then built by including two additional residues along the main-chain, both upstream and downstream of each contact residue. Any overlapping elements are concatenated into single segments. Thus, a descriptor is typically built of several disjoint pieces of the main chain (Fig. 2). It reflects approximately the range of local, most significant physico-chemical interactions between its central residue and surrounding amino-acids. This constitutes a significant difference compared to single segments so frequently used in other studies. Single segments reflect features along the main-chain exclusively, while descriptors are spatial, and thus add a three-dimensional context to local properties of a protein molecule.

In the preliminary stage of computing all pairs of similar descriptors belonging to compared structures are identified. Such pairs constitute small, local pairwise alignments, which can be viewed as building blocks for a larger alignment. In the following stages only alignments which comprise such descriptor pairs are considered. It has already been established, that this approach yields remarkably accurate (also in terms of low RMSD) pairwise alignments (Daniluk and Lesyng, 2011b).

The main difficulty in building a multiple alignment from descriptor alignments results from the fact, that contrary to the pairwise case, it is not enough to select a set of descriptor pairs in which every two of them form a valid alignment. This condition does not prevent conflicts of a kind mentioned above. Therefore, an approach based on computing maximal cliques cannot be straightforwardly used in this case [It would be possible to search for maximal cliques, and then try to resolve all conflicts. However, the problem of resolving conflicts constitutes the most difficult (and intractable) part of the multiple alignment. Furthermore, because resolving conflicts shrinks a clique, such a method would have to take into consideration all maximal cliques, not just the largest ones.].

We have overcome this problem by building an alignment incrementally. If we divide the set of structures into two sets, an optimal multiple alignment is an optimal alignment of multiple alignments of these subsets (Alignments of these subsets do not have to be optimal.). Aligning two multiple alignments may lead to conflicts as well, but their number is expected to be low, when similarity within these alignments is high. This principle leads to an application of a neighbor join (NJ) method. We use a randomized version of the NJ algorithm to generate a set of specimens to be further improved by an evolutionary algorithm.

## 3 Materials and methods

### 3.1 Local descriptors of protein structure

Descriptors have already been applied in several studies (Björkholm *et al.*, 2009; Daniluk and Lesyng, 2011b, 2014; Drabikowski *et al.*, 2007; Hvidsten *et al.*, 2003, 2009; Strömbergsson *et al.*, 2006, 2008). Here, we use a version of the local descriptor methodology described in (Daniluk and Lesyng, 2011a,b, 2014). Every descriptor is built around its central residue. It contains residues that are in contact with the central residue (i.e. $d_{\alpha }\leq 6.5Å$, or $d_{C}\leq 8Å$ and $d_{\alpha }-d_{C}\geq 0.75Å$, where $d_{\alpha }$ and *d*_C_ denote distances between $C_{\alpha }$ atoms and geometrical centers of side-chains, respectively). In the second step, elements around selected residues are built by taking four sequential neighbors, two on each side. Finally, overlapping elements are merged into segments. For more details, see Daniluk and Lesyng (2011a, 2014).

It should be noted that this kind of similarity is totally sequence-independent. Because elements are the smallest indivisible blocks, it is possible that one segment will be aligned to two smaller ones which are a few residues apart.

### 3.2 Pairwise alignments

Once a set of pairs of similar descriptors is computed, one can define a graph, where nodes correspond to descriptor pairs. It can be easily proven, that the largest alignment of two structures corresponds to a maximal clique in such a graph.

Cliques in the graph are identified using a heuristic approach based on the Motzkin-Straus theorem by iteratively searching for a maximum of a certain quadratic form which corresponds to the largest clique in the graph (Daniluk *et al.*, 2019). Possible conflicts are identified and then resolved by a branch-and-bound algorithm searching for a set of descriptor pairs which removal should result in the smallest possible reduction of the alignment size.

### 3.3 Stochastic generation of initial multiple alignments

We incorporated the progressive alignment method to generate a starting population for the evolutionary algorithm. In order to obtain several such alignments, we have developed a process of randomly generating guide trees. We use a method akin to the NJ algorithm, where at each step a pair of clusters with the highest average similarity of their elements is joined. In our implementation, a pair to be joined is chosen randomly with a probability proportional to the average similarity of elements.

### 3.4 Evolutionary algorithm

Multiple alignments generated in a stochastic progressive alignment phase are refined using an evolutionary algorithm. It is an application of a generic strategy mimicking evolutionary processes by maintaining a *population* of *specimens* that correspond to solutions to a problem.**Mutation and crossover**

In the case of mutation, an internal node of a spanning tree is randomly selected and a multiple alignment connected with it is recomputed as a pairwise alignment of multiple alignments in its children. The process is later repeated for all nodes on a path from a selected node to the root of a tree.

In the crossover procedure, structures are randomly divided into two subsets and subalignments containing these subsets are extracted from the chosen specimens. These subalignments are then aligned to obtain a new specimen. A pair of structures is chosen with probability proportional to their distance in spanning trees. They form centroids of subsets for both specimens. After that, structures close to respective centroids in spanning trees are added to subsets. If the process stops before all structures are exhausted, new centroids are selected and iteration is resumed.**Steady-state algorithm**

We applied a steady-state evolutionary algorithm (Whitley *et al.*, 1989). It is performed independently for each specimen immediately after its generation. In this manner, successful individuals can contribute to the new population without an unnecessary delay. To achieve quick convergence, we used a variant of elitist selection. A new specimen is preserved (added to population), if its fitness exceeds the fitness of an individual most similar to it, or if the population has not reached its maximal size. All individuals whose identity to the newly added specimen exceeds 80% are removed from the population.**Gradual extension of the search space**

The evolutionary algorithm starts with refining the consensus of pairwise alignments. After converging, the most under-performing structure is identified by comparing its contribution to the score of the best multiple alignment with scores of its pairwise alignments. This structure is freed by including all descriptor pairs in which one descriptor belongs to this structure in the set of allowed similarities. Then the evolutionary algorithm is restarted. The process is repeated until all structures become unconstrained.

### 3.5 Measure of the alignment size and quality

To evaluate the global quality we assess the spatial arrangement of the local components. We enumerate all pairs of the aligned residues which are in contact in at least one of the aligned structures. Then for each such contact, we compute the RMSD of the respective five residue pieces (elements) of the backbone. These distances are averaged for each residue over all its contacts, for each pair of structures, and after raising to the power of two for the whole multiple alignment. The result can be viewed as an average ‘tension’ exerted on structures when superimposed structures are treated as elastic objects.

Sometimes a pairwise alignment can be divided into regions with no contacts between them. In such a case, possible conformational distortions would not influence tension. Therefore, we augment the score of all regions, except the largest one, by a factor proportional to $\sqrt{\frac{1+\text{cos}\;\alpha }{2}}$, where *α* is an angle between rotations required to superimpose the largest region and the augmented one, respectively.

### 3.6 Core alignment and its refinement

We use a two-stage process similar to the one used by our DEDAL method (Daniluk and Lesyng, 2011b). An optimal multiple alignment is built using only descriptor alignments that have at least three segments. Such alignment in principle contains all similarities of the protein cores, but may not cover loops and extended linkers. In the second stage, the algorithm is rerun to extend computed alignment with all remaining descriptor pairs which are consistent and overlap the alignment computed in the first stage.

## 4 Implementation

We have implemented the described algorithm in C on the Linux platform. For the rapid finding of cliques in large graphs we have used CUDA-MS—a GPU accelerated library (Daniluk *et al.*, 2019).

We have made DAMA available online at http://dworkowa.imdik.pan.pl/EP/DAMA. Linux binaries of the software are available upon request. The server can be used to align structures identified by PDB or SCOP accession codes or supplied in uploaded files. Superpositions can be downloaded as PDB files and also viewed through the WebGL applet. The alignments are available in FASTA format and as a list of corresponding residue ranges.

Scalability of implementation was shown in Supplementary Figure S3 in Supplementary Materials.

## 5 Results

### 5.1 SISY-multiple dataset

In this study, we have used SISY-multiple—a set of multiple alignments created especially for assessment of the quality of multiple structure alignment methods (Berbalk *et al.*, 2009). It is based on SISYPHUS—a manually curated set of multiple structure alignments (Andreeva *et al.*, 2007), which has been pruned from ambiguities. It contains 106 multiple alignments comprising from 3 to 119 structures (∼13 on average). Several of them contain particular difficulties such as repetitions, insertions/deletions, permutations or conformational variabilities. This set has been used for testing several alignment algorithms already.

Berbalk *et al.* use two measures to assess the similarity of a given alignment to a reference one. A more stringent measure (*Q*_C_) is the ratio of correctly aligned columns, while a more lenient one (*Q*_P_) is the ratio of correctly aligned residue pairs. It should be noted, that all alignments in SISY-multiple have all columns completely filled (i.e. contain residues belonging to a core common to all structures). We present values of *Q*_C_ and *Q*_P_ for alignments computed by Caretta (Akdel *et al.*, 2020), MASS (Dror *et al.*, 2003), Matt (Menke *et al.*, 2008), MultiProt (Shatsky *et al.*, 2004), MUSTANG (Konagurthu *et al.*, 2006), POSA (Ye and Godzik, 2005), 3DCOMB (Wang *et al.*, 2011), MISTRAL (Micheletti and Orland, 2009), MAMMOTH (Lupyan *et al.*, 2005), MAPSCI (Ilinkin *et al.*, 2010), mTM-align (Dong *et al.*, 2018) and DAMA. The results for the first five methods are taken from (Berbalk *et al.*, 2009). For the remaining ones, we have performed computations ourselves. Several methods fail in some cases either due to internal faults or incompatibility of input data (MUSTANG, Matt and POSA are incapable of aligning structures with multiple chains). In his study Berbalk *et al.* have chosen a set of 61 alignments for which no program has failed.

**Fig. 3.**
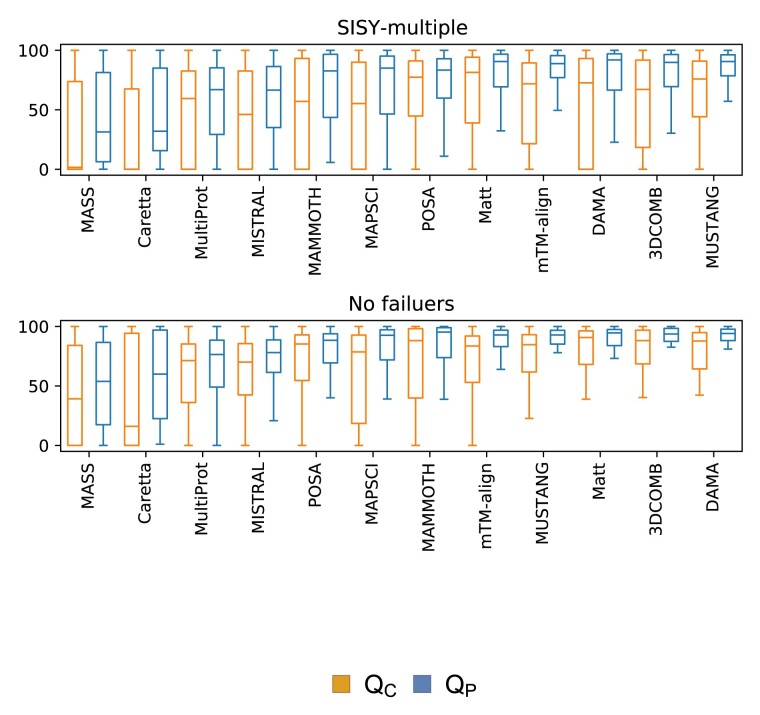
Accuracy on the SISY-multiple dataset for the *Q*_C_ and *Q*_P_ measures. The upper panel contains results for all alignments, while the lower panel contains results only for alignments for which none of the methods have failed

DAMA turned out to be the most accurate method on the whole SISY-multiple dataset achieving median accuracy of 82.3% for the *Q*_C_ and 92.7% for *Q*_P_ measure, second-best 3DCOMB achieved 67.1% and 89.8% respectively (67.6% and 89.9% when limited to cases for which program has not failed). If one disregards cases for which programs have failed, Matt (*Q*_C_: 81.4%, *Q*_P_: 90.6%), POSA (*Q*_C_: 77.4%, *Q*_P_: 88.3%) and MUSTANG (*Q*_C_: 75.9%, *Q*_P_: 90.6%) perform better than 3DCOMB. However, these three methods have the highest number of failures. Results for the remaining methods are provided in Table 1 and Figure 3.

**Table 1.** Median values of *Q*_C_ and *Q*_P_ measures for the tested methods on the whole SISY-multiple set (106 alignments) and excluding failures for each method (60 alignments)

	Whole set		No failures		Failures
	*Q _C_*	*Q*_P_	*Q*_C_	*Q*_P_	
MASS	0.00	23.44	1.59	31.38	13
Matt	58.48	83.58	81.41	90.59	22
MultiProt	55.21	65.50	59.48	66.94	4
MUSTANG	52.43	82.07	75.86	90.58	31
POSA	58.49	76.94	77.36	83.33	19
3DCOMB	67.14	89.83	88.12	93.66	0
mTM-align	71.78	88.80	83.56	92.81	0
Caretta	00.00	31.59	16.09	59.91	0
MISTRAL	42.56	64.79	70.06	78.02	4
MAMMOTH	53.57	81.05	88.10	95.21	4
MAPSCI	55.25	85.01	78.56	92.57	0
DAMA	72.68	91.93	87.78	94.07	0

**Fig. 4.**
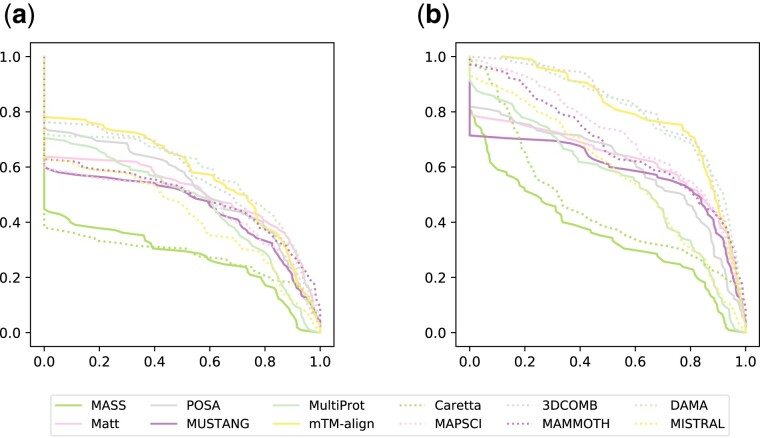
Performance profiles for all alignments in the SISY-multiple dataset for the *Q*_C_ (**a**) and *Q*_P_ (**b**) measures

Performance profiles (Dolan and Moré, 2002) are convenient to assess quality on a large dataset. In this case, however, we used them to compare the accuracy of the algorithms tested. Let $c_{m,a}$ be the accuracy of the solution computed by the method *m* for the alignment *a—*with either the *Q*_C_ or *Q*_P_ measures. We define accuracy ratio as $r_{m,a}=\frac{c_{m,a}}{\max_{m}c_{m,a}}$. These ratios are aggregated into profiles for each method:

$$\rho_{m}(\alpha )=\frac{\left|a\in A:r_{g,a}\geq \alpha \right|}{\left|A\right|}$$

where A is a set of reference alignments, and |·| denotes the set cardinality. According to Dolan and Moré performance profiles may be interpreted as probabilities for a method to achieve performance not worse than the best method by a given ratio. Performance profiles for all alignments in the SISY-multiple dataset for the *Q*_C_ (a) and *Q*_P_ (b) measure are presented in Figure 4, and profiles for the subset of 61 safe alignments selected from the SISY-multiple dataset by Berbalk *et al.* are presented in Supplementary Figure S2 in Supplementary Materials. Performance profiles show that DAMA is the most likely method to retain a satisfactory alignment regardless of the desired accuracy. MUSTANG, Matt and POSA along with 3DCOMB perform very well on the subset of easier alignments. However, when compared using the whole set, only 3DCOMB and DAMA remain outstanding. There is a significant difference between the *Q*_C_ and *Q*_P_ profiles for these methods indicating that DAMA is more likely to align whole columns correctly, and thus identify the whole common core. The numerical values of AUC are provided in Supplementary Table S3 in Supplementary Materials.

Sample alignment from the SISY-multiple dataset returned by DAMA (containing circular permutation) can be found in Supplementary Table S6 in Supplementary Materials.

### 5.2 Case study: protein kinases

As reference, we have taken an alignment of 31 kinases prepared by Scheeff and Bourne (Scheeff and Bourne, 2005). This alignment includes 25 typical protein kinases (TPKs) and 6 atypical kinases (AKs). The authors describe 20 features characteristic to some kinase families or of all of them (see Supplementary Tables S1 and S2 in Supplementary Materials). We have identified 240 aligned positions in that curated multiple alignment corresponding to notable features, and used them to test two methods performing best on the SISY-multiple set—3DCOMB and DAMA.

#### 5.2.1 Highly conserved residues

**Fig. 5.**
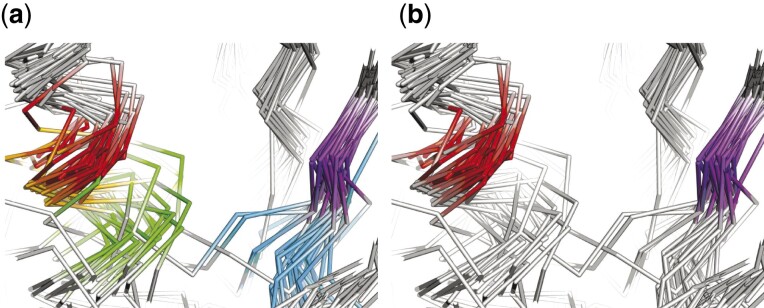
Alignment of two conserved residues K72 and E91 [as noted by (Scheeff and Bourne, 2005)] by 3DCOMB (**a**) and DAMA (**b**). Structural elements corresponding to columns in alignment are indicated with different colors

There are few residues playing important role in kinase activity which are conserved in all structures, in particular K72, E91, D166 or D184 [positions according to PKA (1cdk)]. Residues crucial for the ATP hydrolysis (D184) and present in the catalytic region (D166) were aligned correctly by both methods. However, only DAMA did equally well with residues responsible for ATP stabilization in the binding pocket (K72 and E91), while 3DCOMB shifted its alignment of three structures by 1–2 residues (see Fig. 5). There is also a number of other residues which are conserved in some structures. Examples are H158 and D220 forming hydrogen bonds stabilizing the catalytic region in most kinases, and N171 or equivalent isoleucine or glutamine interacting with an ${\text{Mg}}^{+2}$ cation important for the catalytic process. Aligning N171 or H158 caused no difficulties. 3DCOMB aligned all residues at position D220, while DAMA shifted respective residues in PDK1, GSK3 and PKB by one position and IRK by three.

#### 5.2.2 Secondary structure

**Fig. 6.**
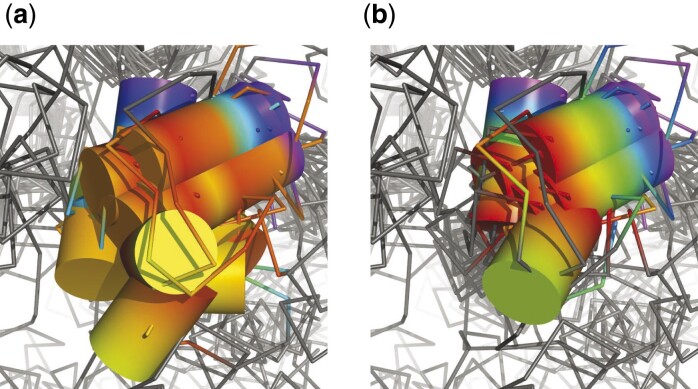
Superposition of the aligned helix B or equivalent loops for alignments yielded by 3DCOMB (**a**) and DAMA (**b**)

There are several secondary structures conserved in the reference alignment. Among them, helices denoted with letters A–F are common for all structures (except for B-helix), while G to I α-helices are present only in TPKs and vary in length. B-helix is shared only by AGC kinases (five out of TPKs) and ChaK, while in other structures a loop takes its place. Also a number of β-strands numbered from 1 to 8 forming a β-sheet N-terminal domain is present in all structures (see Fig. 6 and Supplementary Fig. S2 in Supplementary Materials).

C- and D-helices or β-strand 4 were aligned correctly for TPKs by both programs. 3DCOMB failed, however, to align corresponding regions in AKs due to incorrect gap placement. Well-aligned remaining secondary structures are F-helix, containing aspartate D220 forming H-bond with H158, common to all kinases, as well as, G- and H-helices present in TPKs exclusively. In the DAMA alignment four shifts occur in F-helix and propagate further through all following helices, but remaining structures are aligned correctly, including AKs F-helix. 3DCOMB, on the other hand, incorrectly aligned F-helix in AKs, but committed no errors in the case of G- and H-helices in TPKs.

The most troublesome elements were helix B (or its substitute loop), and α-helix I common for all TPKs. 3DCOMB aligned correctly only half of I helices, applying minor shifts in the remaining structures, while the result from DAMA shows only shifts in four previously mentioned structures. Helix B, on the other hand, was aligned correctly by DAMA, while 3DCOMB found only two correct pairs out of thirty (partially aligning remaining pairs).

#### 5.2.3 Insertions

**Fig. 7.**
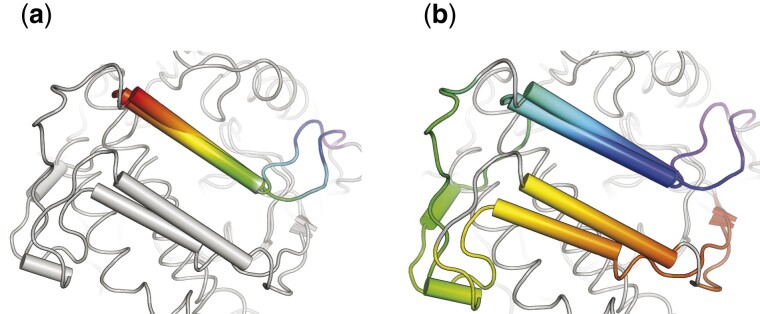
Comparison of the aligned loops from CKA-2 and APH(3′)-IIIa. AKs obtained from 3DCOMB (**a**) and DAMA (**b**). Aligned residues are colored the same way

There are four insertions present only in some kinases. One present in the catalytic region of one structure—AFK—was not aligned with any residues from other structures by both programs as expected. Long insertions in CKA-2 and APH were correctly aligned by DAMA, and 3DCOMB achieved approximately 25% accuracy (see Fig. 7). The similarity of insertions between G- and H-helices shared by CMGC kinases was detected by DAMA in 3 out of 5 structures, while 3DCOMB failed to align any inserted regions in these structures. In the case of insertion preceding I-helix, shared by five ACG kinases, DAMA missed one structure, while 3DCOMB detected similarity for only one pair of structures.

#### 5.2.4 Other

Summary of all identified structural features and performance of DAMA and 3DCOMB can be found in Supplementary Tables S4 and S5 in Supplementary Materials.

## 6 Conclusion

DAMA is capable of computing multiple alignments of large sets of structures, imposing only minor constraints in the process. It is based on local similarities, which may comprise several disjoint segments of a protein backbone and encompass complete physico-chemical neighborhoods of amino-acid residues. It is constrained neither by protein topology, thus permitting segments swaps and circular permutations, nor by its global structure allowing for conformational variability which, in particular, is essential for enzymatic and other activities.

In our approach, the alignment algorithm searches for the largest non-contradictory ensemble of similar descriptors. Local descriptors are generic enough to capture most of the biologically significant similarities present in the test set, while at the same time they are discriminatory enough to prevent the emergence of larger, but meaningless, solutions. This result is consistent with our previous study demonstrating the applicability of local descriptors to the pairwise alignment problem (Daniluk and Lesyng, 2011b, 2014).

When solving a so-called black-box optimization problem, in which an optimized function (in this case the alignment score) cannot be differentiated and has to be independently evaluated for each attempted solution, it is crucial to limit the number of unsuccessful trials by reducing the number of infeasible solutions. It has been established that the multiple alignment problem is NP-complete (Daniluk and Lesyng, 2014), and thus the development of an accurate algorithm with polynomial time complexity is highly unlikely. However, the DAMA example shows, that an effective heuristic approach can be developed without imposing artificial restrictions (e.g. lack of segment swaps, global RMSD threshold) which would limit the solution space.

3DCOMB, the second-best method after DAMA tested in this study, uses a similar definition of local similarities—so-called local and global structure environments. Its search space is subject to the aforementioned constraints due to the usage of dynamic programming and TM-score (Zhang and Skolnick, 2004) for assessment of the resulting alignments. This gives 3DCOMB an advantage over DAMA in easy cases. In several cases, these simplifying assumptions make the correct alignment infeasible which causes 3DCOMB to perform slightly worse.

The main goal to compute multiple alignments is not only to assess whether given structures are similar, but also to discover the exact nature of this similarity. Such an aim can be achieved only if an alignment algorithm reliably reconstructs whole columns of the optimal alignment. The test performed on protein kinases shows DAMA’s capability in this respect. Performance profiles (see Fig. 4 and Supplementary Fig. S1 in Supplementary Material) also show that advantage of DAMA over other methods are more pronounced when using the *Q*_C_ measure.

## Funding

This study was supported by the Z-526/22 [IMDiK PAN] and PP/BF-501-D111-01-1110102 (UW) funds. Computations were carried out using the infrastructure funded by the POIG.02.03.00-00-003/09 (Biocentrum-Ochota) and POIG.02.01.00-14-122/09 (Physics at the basis of new technologies) projects.

*Conflict of Interest*: none declared.

## Supplementary Material

btab571_Supplementary_Data
