# Supplementary material for: DAMA: a method for computing multiple alignments of protein structures using local structure descriptors
Source: Bioinformatics. 2021 Aug 16;38(1):80–5. doi: 10.1093/bioinformatics/btab571 (PMC8696102; doi:10.1093/bioinformatics/btab571)
Supplement: btab571_Supplementary_Data [file btab571_supplementary_data.pdf]

Table S1: A subset of the protein kinase type superfamily used in the structural alignment validation study of DAMA.

PDB id	Group	Name	Description
1BO1	ATK	PIPKIIβ	Type IIβ phospatidylinositol phosphate kinase
1IA9	ATK	ChaK	Transient receptor potential channel kinase domain
1E8X	ATK	P13K	Phosphoinositide 3-kinase catalytic subunit
1CJA	ATK	AFK	Actin-fragmin kinase
1NW1	ATK	CKA-2	Choline Kinase
1J7U	ATK	APH(3')-IIIa	Aminoglycoside phosphotransferase
1CDK	AGC	PKA	cAMP dependent protein kinase
1O6L	AGC	PKB/Akt	Protein kinase B
1OMW	AGC	GRK2	G protein-coupled receptor kinase 2
1H1W	AGC	PDK1	3-phosphoinositide dependent protein kinase-1
1MUO	Other	Aur2	Aurora-2 kinase
1TKI	CAMK	TK	Titin kinase
1JKL	CAMK	DAPK	Death-associated protein kinase
1A06	CAMK	CaMK1	Calcium/calmodulin-dependent protein kinase 1
1PHK	CAMK	PhK	Phosphorylase kinase
1KWP	CAMK	MAPKAPK2	Mitogen-activated protein kinase-activated protein kinase 2
1IA8	CAMK	Chk1	Cell cycle checkpoint kinase
1GNG	CMGC	GSK3	Glycogen synthase kinase 3
1HCK	CMGC	CDK2	Cyclin-dependent kinase 2
1JNK	CMGC	JNK3	c-Jun N-terminal kinase 3
1HOW	CMGC	Sky1p	–
1LP4	Other		Protein kinase CK2
1F3M	STE	PAK1	p21-activated kinase 1
1O6Y	Other	PknB	–
1CSN	CK1	CK1	Casein kinase 1
1B6C	TKL	TGFβR1	Type 1 TG93 receptor
2SRC	TK	c-Src	–
1LUF	TK	MuSK	Muscle-specific kinase
1IR3	TK	IRK	Insulin receptor kinase
1M14	TK	EGFR	Epidermal growth factor receptor kinase domain
1GJO	TK	FGFR2	Fibroblast growth factor receptor 2 kinase domain

Table S2: Structural features used to analyze the structural alignments of the selected kinase-like proteins - compare to Scheeff and Bourne [2005].

No.	Feature
1	Ion pair analogous to K72-E91 in PKA.
2	α-Helix B.
3	α-Helix C.
4	β-strand 4.
5	Helical structure in the area of α-Helix D.
6	α-Helix E.
7	Key conserved histidine at H158.
8	Large helical insertion between Helix E and Strand 6.
9	Structure underlying the catalytic region.
10	Architecture of the catalytic region.
11	Insertion in the catalytic region.
12	Asp residue at 171, or apparent compensation for its absence.
13	Similar direct hydrophobic link between catalytic region and Helix E.
14	Nature of structure linking Strand 9 and Helix F / analogous ion pair in PKA.
15	Helix F.
16	Helices G, H and I.
17	Extensive helical insertions between Helix G and Helix H (AGCs only).
18	Insertion between R280 and Helix I (CMGCs only).
19	Helix I structure (TPKs only).

Table S3: Area under the profile curves shown in Figures 4 and S1.

	Whole set		Safe subset	
	Q_C	Q_P	Q_C	Q_P
MASS	0.300	0.372	0.475	0.540
Matt	0.584	0.660	0.845	0.899
MultiProt	0.526	0.573	0.647	0.677
MUSTANG	0.517	0.620	0.798	0.899
POSA	0.615	0.631	0.814	0.814
3DCOMB	0.680	0.866	0.842	0.938
MISTRAL	0.478	0.607	0.675	0.736
MMult	0.551	0.723	0.741	0.864
MAPSCI	0.525	0.753	0.696	0.829
DAMA	0.771	0.908	0.849	0.946

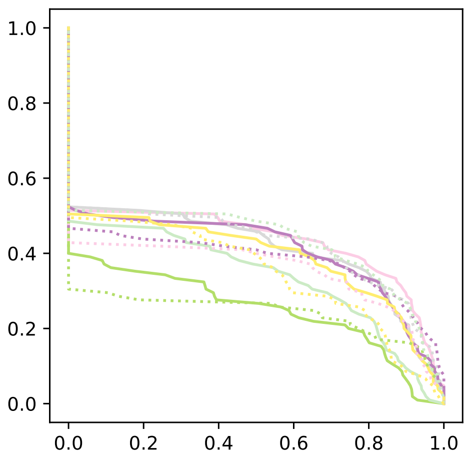

(a)

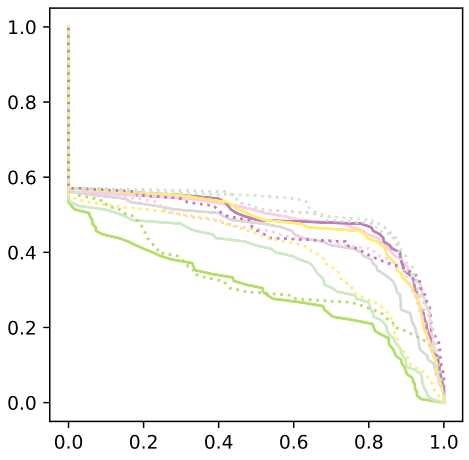

(b)

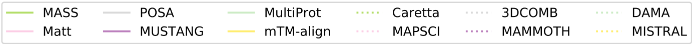

Figure S1: Performance profiles for the subset of 60 alignments selected from the SISY-multiple dataset, for which none of the methods failed, for the Q_C (a) and Q_P (b) measures.

Table S4: Agreement of the 3DCOMB alignment with the reference set concerning 19 traits enumerated in [Scheeff and Bourne, 2005]. Numbers denote classes of the trait alignments. Structures belonging to the same class are aligned with each other according to reference mentioned above. Note, that a method reproducing reference alignment would result in only one class for each trait. Blanks indicate lack of traits.

Structure ID	Trait																		
	1	2	3	4	5	6	7	8	9	10	11	12	13	14	15	16	17	18	19
1jkl A	1	10	1	1	1	1	1		1	1		1	1	2	1	1			1
1o6l A	1	3	1	1	1	1	1		1	1		1	1	17	1	1	5		1
1m14 A	1	2	1	1	1	1	1		1	1		1	1	15	1	1			6
1phk A	1	8	1	1	1	1	1		1	1		1	1	2	1	1			1
1tki A	1	1	1	1	1	1	1		1	1		1	1	3	1	1			1
1luf A	1	2	1	1	1	1	1		1	1		1	1	10	1	1			5
1jnk A	1	9	1	1	1	1	1		1	1		1	1	6	1	1		3	1
1ir3 A	1	2	3	1	1	1	1		1	1		1	1	5	1	1			4
1csn A	1	11	2	1	1	1	1		1	1		1	1	21	1	1			2
1hck A	3	4	4	1	1	1	1		1	1		1	1	19	1	1		5	1
2src A	1	1	1	1	1	1	1		1	1		1	1	16	1	1			2
1how A	1	1	1	1	1	1	1		1	1		1	1	4	1	1		1	1
1o6y A	1	6	1	1	1	1	1		1	1		1	1	3	1	1	1		2
1cdk A	1	3	1	1	1	1	1		1	1		1	1	18	1	1	2		1
1omw A	4	5	6	1	1	1	1		1	1		1	1	20	1	1	3		1
1a06 A	1	1	2	1	1	1	1		1	1		1	1	3	1	1			1
1kwp A	1	1	1	1	1	1	1		1	1		1	1	4	1	1			1
1muo A	1	5	1	1	1	1	1		1	1		1	1	1	1	1			1
1h1w A	1	3	1	1	1	1	1		1	1		1	1	2	1	1	4		1
1lp4 A	1	1	1	1	1	1	1		1	1		1	1	2	1	1		4	3
1ia8 A	1	4	1	1	1	1	1		1	1		1	1	7	1	1			1
1b6c B	1	1	1	1	1	1	1		1	1		1	1	13	1	1			2
1f3m C	1	3	1	1	1	1	1		1	1		1	1	12	1	1			1
1gng A	1		1	1	1	1	1		1	1		1	1	9	1	1		2	1
1gjo A	1	2	1	1	1	1	1		1	1		1	1	8	1	1			1
1bo1 A	1		1	1	1	1	1		2	2		2	2	14	3				
1ia9 A	1	7	1	1		1	1		1	2		1	1	11	2				
1e8x A	2		2	2	1	3	1		4	1		1	1		5				
1cja A	1		1	2	2	2	1		3	1	1	1	1	1	4				
1nw1 A	1	1	1	3	3	2	1	1	1	1		1	1	1	2				
1j7u A	1	1	5	1	1	2	1	2	1	1		1	1	1	2				

Table S5: Agreement of the DAMA alignment with the reference set concerning 19 traits enumerated in [Scheeff and Bourne, 2005]. Numbers denote classes of the trait alignments. Structures belonging to the same class are aligned with each other according to reference mentioned above. Note, that a method reproducing reference alignment would result in only one class for each trait. Blanks indicate lack of traits.

Structure ID	Trait																		
	1	2	3	4	5	6	7	8	9	10	11	12	13	14	15	16	17	18	19
1jkl A	1	1	1	1	1	1	1		1	1		1	1	1	1	1			1
1o6l A	1	1	1	1	1	1	1		2	1		1	1	2	2	2	2		2
1m14 A	1	1	1	1	1	1	1		1	1		1	1	1	1	1			1
1phk A	1	1	1	1	1	1	1		1	1		1	1	1	1	1			1
1tki A	1	1	1	1	1	1	1		1	1		1	1	1	1	1			1
1luf A	1	1	1	1	1	1	1		1	1		1	1	1	1	1			1
1jnk A	1	1	1	1	1	1	1		1	1		1	1	1	1	1		1	1
1ir3 A	1	1	1	1	1	1	1		3	1		1	1	3	3	3			3
1csn A	1	1	1	1	1	1	1		1	1		1	1	1	1	1			1
1hck A	1	1	1	1	1	1	1		1	1		1	1	1	1	1		1	1
2src A	1	1	1	1	1	1	1		1	1		1	1	1	1	1			1
1how A	1	1	1	1	1	1	1		1	1		1	1	1	1	1		1	1
1o6y A	1	1	1	1	1	1	1		1	1		1	1	1	1	1	1		1
1cdk A	1	1	1	1	1	1	1		1	1		1	1	1	1	1	1		1
1omw A	1	1	1	1	1	1	1		1	1		1	1	1	1	1	1		1
1a06 A	1	1	1	1	1	1	1		1	1		1	1	1	1	1			1
1kwp A	1	1	1	1	1	1	1		1	1		1	1	1	1	1			1
1muo A	1	1	1	1	1	1	1		1	1		1	1	1	1	1			1
1h1w A	1	1	1	1	1	1	1		2	1		1	1	2	2	2	3		2
1lp4 A	1	1	1	1	1	1	1		1	1		1	1	1	1	1		1	1
1ia8 A	1	1	1	1	1	1	1		1	1		1	1	1	1	1			1
1b6c B	1	1	1	1	1	1	1		1	1		1	1	1	1	1			1
1f3m C	1	1	1	1	1	1	1		1	1		1	1	1	1	1			1
1gng A	1		1	1	1	1	1		2	1		1	1	2	2	2		2	2
1gjo A	1	1	1	1	1	1	1		1	1		1	1	1	1	1			1
1bo1 A	1		1	1	1	1	1		1	1		1	1	1	1				
1ia9 A	1	1	1	1		1	1		1	1		1	1	1	1				
1e8x A	1		1	1	1	1	1		1	1		1	1		1				
1cja A	1		1	1	1	1	1		1	1	1	1	1	2	1				
1nw1 A	1	1	1	1	1	1	1	1	1	1		1	1	2	1				
1j7u A	1	1	1	1	1	1	1	1	1	1		1	1	2	1				

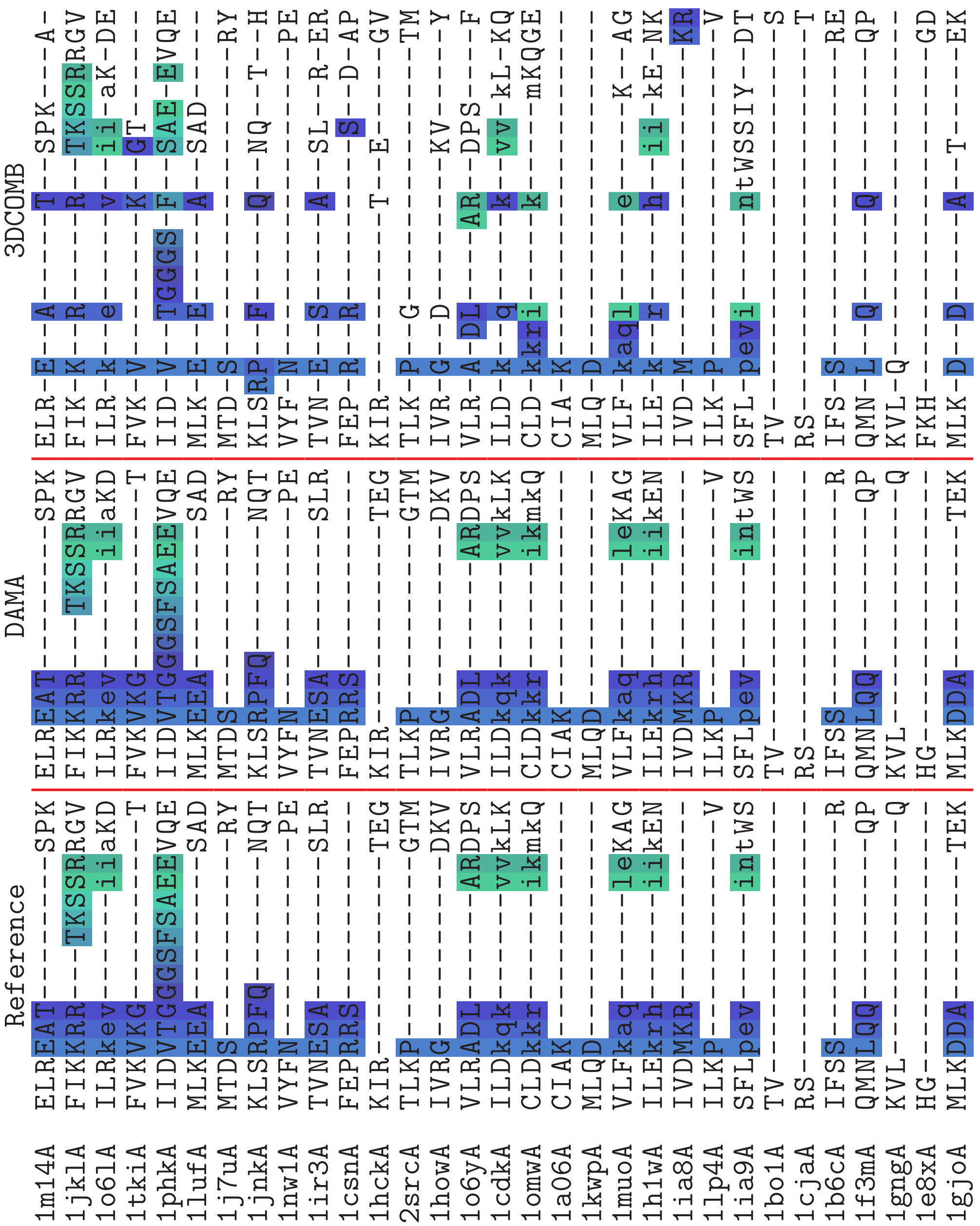

Figure S2: Alignment of helices B or equivalent loops yielded by 3DCOMB and DAMA. Reference by Scheeff and Bourne (2005)

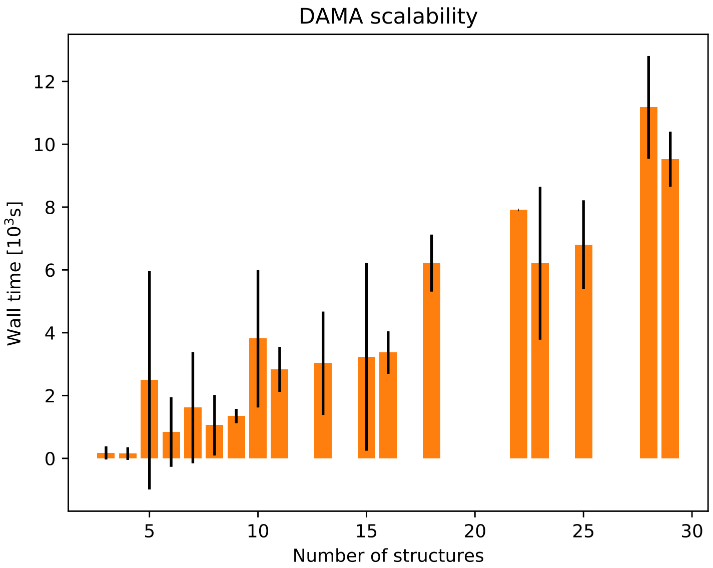

Figure S3: Scalability of DAMA measured on SISY-multiple. Bars show mean wall time for alignment of the same number of structures (with less than 30 structures) in SISY-multiple data set. Error bars show standard deviation. Calculation were preformed on nodes equipped with 2x Intel Xeon E5-2640 2.50 GHz CPU and GeForce GTX 680 GPUs

Table S6: Alignment AL00047861 from SISY-Multiple resolved by DAMA. As reported in SISY-Multiple dataset description – curated manual alignment contains structural circular permutation and corformational variability.

1l9lA	1m12A	1n69B	1nklA	1of9A	1qdmA
A:1:G	A:2:D	-	A:1:G	-	A:66S:D
A:2:R	A:3:V	B:2:D	A:2:Y	A:3:I	A:67S:P
A:3:D	A:4:Y	B:3:V	A:3:F	A:4:L	A:68S:M
A:4:Y	A:5:C	B:4:C	A:4:C	A:5:C	A:69S:C
A:5:R	A:6:E	B:5:Q	A:5:E	A:6:N	A:70S:S
A:6:T	A:7:V	B:6:D	A:6:S	A:7:L	A:71S:A
A:7:C	A:8:C	B:7:C	A:7:C	A:8:C	A:72S:C
A:8:L	A:9:E	B:8:I	A:8:R	A:9:T	A:73S:E
A:9:T	A:10:F	B:9:Q	A:9:K	A:10:G	A:74S:M
A:10:I	A:11:L	B:10:M	A:10:I	A:11:L	A:75S:A
A:11:V	A:12:V	B:11:V	A:11:I	A:12:I	A:76S:V
A:12:Q	A:13:K	B:12:T	A:12:Q	A:13:N	A:77S:V
A:13:K	A:14:E	B:13:D	A:13:K	A:14:T	A:78S:W
A:14:L	A:15:V	B:14:I	A:14:L	A:15:L	A:79S:M
A:15:K	A:16:T	B:15:Q	A:15:E	A:16:E	A:80S:Q
A:16:K	A:17:K	B:16:T	A:16:D	A:17:N	A:81S:N
A:17:M	A:18:L	B:17:A	A:17:M	A:18:L	A:82S:Q
A:18:V	A:19:I	B:18:V	A:18:V	A:19:L	A:83S:L
A:19:D	A:20:D	B:19:R	A:19:G	A:20:T	A:84S:A
A:20:K	A:21:N	B:20:T	A:20:P	-	A:85S:Q
-	A:22:N	B:21:N	A:21:Q	A:21:T	A:86S:N
A:21:P	A:23:K	-	A:22:P	A:22:K	A:87S:K
A:22:T	A:24:T	-	A:23:N	A:23:G	A:88S:T
A:23:Q	A:25:E	B:22:S	A:24:E	A:24:A	A:89S:Q
A:24:R	A:26:K	B:23:T	A:25:D	A:25:D	A:90S:D
A:25:S	A:27:E	B:24:F	A:26:T	A:26:K	A:91S:L
A:26:V	A:28:I	B:25:V	A:27:V	A:27:V	A:92S:I
A:27:S	A:29:L	B:26:Q	A:28:T	A:28:K	A:93S:L
A:28:N	A:30:D	B:27:A	A:29:Q	A:29:D	A:94S:D
A:29:A	A:31:A	B:28:L	A:30:A	A:30:Y	A:95S:Y
A:30:A	A:32:F	B:29:V	A:31:A	A:31:I	A:96S:V
A:31:T	A:33:D	B:30:E	A:32:S	A:32:S	A:97S:N
A:32:R	A:34:K	B:31:H	A:33:Q	A:33:S	A:98S:Q
A:33:V	A:35:M	B:32:V	A:34:V	A:34:L	A:99S:L
A:34:C	A:36:C	B:33:K	A:35:C	A:35:C	A:100S:C
A:35:R	A:37:S	B:34:E	A:36:D	A:36:N	A:101S:N
A:36:T	A:38:K	B:35:E	A:37:K	A:37:K	A:102S:R
A:37:G	A:39:L	-	A:38:L	A:38:A	A:103S:L
A:39:S	A:40:P	-	A:39:K	A:40:G	A:104S:P
A:40:R	A:41:K	-	A:40:I	A:41:F	A:248:S

Table S6 – continuation

-	A:42:S	-	-	-	A:249:P
A:41:W	A:43:L	-	A:41:L	A:42:I	-
A:42:R	A:44:S	-	A:42:R	A:43:A	A:3S:S
A:43:D	A:45:E	-	A:43:G	A:44:T	A:4S:Q
A:44:V	A:46:E	-	A:44:L	-	A:5S:E
A:45:C	A:47:C	-	A:45:C	A:45:L	A:6S:C
A:46:R	A:48:Q	-	A:46:K	A:46:C	A:7S:K
A:47:N	A:49:E	B:49:N	A:47:K	A:47:T	A:8S:T
A:48:F	A:50:V	B:50:Y	A:48:I	A:48:K	A:9S:I
A:49:M	A:51:V	B:51:I	A:49:M	A:49:V	A:10S:V
A:50:R	A:52:D	B:52:S	A:50:R	A:50:L	A:11S:S
A:51:R	A:53:T	B:53:Q	A:51:S	A:51:D	A:12S:Q
A:52:Y	A:54:Y	B:54:Y	A:52:F	A:52:F	A:13S:Y
A:53:Q	A:55:G	B:55:S	A:53:L	A:53:G	A:14S:G
A:54:S	A:56:S	B:56:E	A:54:R	A:55:D	A:15S:Q
A:55:R	A:57:S	B:57:I	A:55:R	A:56:K	A:16S:Q
A:56:V	A:58:I	B:58:A	A:56:I	A:57:L	A:17S:I
A:57:I	A:59:L	B:59:I	A:57:S	A:58:I	A:18S:L
A:58:Q	A:60:S	B:60:Q	A:58:W	A:59:Q	A:19S:D
A:59:G	A:61:I	B:61:M	A:59:D	A:60:L	A:20S:L
A:60:L	A:62:L	B:62:M	A:60:I	A:61:I	A:21S:L
A:61:V	A:63:L	B:63:M	A:61:L	A:62:E	A:22S:L
A:62:A	A:64:E	B:64:H	A:62:T	A:63:D	A:23S:A
A:63:G	A:65:E	-	A:63:G	A:64:K	A:24S:E
A:64:E	A:66:V	B:65:M	A:64:K	A:65:V	A:25S:T
A:65:T	A:67:S	B:66:Q	A:65:K	A:66:D	A:26S:Q
A:66:A	A:68:P	B:67:P	A:66:P	A:67:A	A:27S:P
A:67:Q	A:69:E	B:68:K	A:67:Q	A:68:N	A:28S:K
A:68:Q	A:70:L	B:69:E	A:68:A	A:69:A	A:29S:K
A:69:I	A:71:V	B:70:I	A:69:I	A:70:I	A:30S:I
A:70:C	A:72:C	B:71:C	A:70:C	A:71:C	A:31S:C
A:71:E	A:73:S	B:72:A	A:71:V	A:72:A	A:32S:S
A:72:D	A:74:M	B:73:L	A:72:D	A:73:K	A:33S:Q
A:73:L	A:75:L	B:74:V	A:73:I	A:74:I	A:34S:V
A:74:R	A:76:H	B:75:G	A:74:K	A:75:H	A:35S:G
-	A:77:L	B:76:F	A:75:I	A:76:A	A:36S:L
-	A:78:C	B:77:C	A:76:C	-	A:37S:C
-	A:79:S	B:78:D	A:77:K	-	-
-	A:80:G	-	A:78:E	-	-
